# Harvard Odontological Society

**Published:** 1900-06

**Authors:** 

**Affiliations:** Harvard Odontological Society


### HARVARD ODONTOLOGICAL SOCIETY.

AT a meeting of the Harvard Odontological Society held in Boston, September 28, 1899, a paper was read by Frank R. Dickerman, D.M.D., of Taunton, Mass., entitled "Some Effects of Immigration from a Dental View."

(For Dr. Dickerman's paper, see page 384.)

DISCUSSION.

*President Clapp.*—We have listened to this very interesting paper on a subject that is somewhat new to us, and consequently is of greater interest on that account. When I read the subject of the paper, I did not have my operating-glasses on, and I read it "Some Effects of the Imagination" instead of "Immigration." Now, we will not charge the doctor anything for the suggestion, but hope he will write us a paper on that subject also.

*Dr. Taylor.*—Perhaps in my position in practice in the city I see as large a proportion of the foreign born and those of foreign parentage as almost any member of the Society. I think I have no real definite statements to make as to the results of immigration, except this one point that has been frequently brought to my attention. People tell me that in the old country (wherever that may be) their teeth were very good; that they did not have any trouble with them at all until after they had been in this country a few years, and their trouble dated from the time of their arrival. If such is the fact, it is very difficult to say to what it may be due. It may be to change of food, or to the fact that their habits of life are very different in this country from what they were in the foreign countries from which they came. Perhaps the largest proportion of those who have complained to me in that way have been young women, mostly of the Irish or Swedish races, and I think largely those who have entered domestic service in this country, having previously been engaged in some form of manual or field labor in the countries from which they came; that is, the kind of labor has been changed from an out-door occupation to an entirely in-door service. At the same time, it is undoubtedly a fact that the peasant classes in the European countries live on a different kind of diet from the people who serve in the kitchens of American families. I think that perhaps both those things have a strong effect on the condition of the teeth. A change from the coarse and comparatively plain food of the old countries to the rather more delicate and highly prepared food of the American kitchen must have quite a good deal to do with the teeth, inasmuch as the teeth are not called upon for so much exercise. It is probably a fact that in the countries from which they came very little care is necessary in order to keep the teeth clean, as the coarse foods that are used and the consequent mastication required have a cleansing effect upon the teeth.

Bearing in mind these facts, of the change in diet, and the entire change in the habits of life, it hardly seems to me that the subject of the effect of immigration upon the teeth ought to be considered, except from the stand-point of the effect of the mingling of races.

*Dr. Blaisdell.*—I think we are overlooking one point in this discussion. Dr. Taylor has referred to the difference in the character of the teeth of a certain class of people,—domestics,—stating that they have changed their modes of life entirely. If we want to make a comparison to show the effect of immigration, we should take a class of people who pursue the same life here that they did before they came to this country. For instance, suppose we take Italian laborers. They come here and do about the same character of work as they did in their old homes, and how often are they in need of dental service? Around Portsmouth we have had in the last four or five years a good many Italian laborers employed in the streets, water-works, etc., and I cannot remember of seeing more than two at the most, and their conditions were due more to accident than decay.

It seems to me that a change in the habits of life would have more to do with a depreciation in the character of the teeth than would the mere fact of immigration to this country.

I have noticed this marked change in regard to the teeth of Norwegian girls, but I do not know the life that they led in their old homes. I have seen a number of those girls who have come to this country at the age of about eighteen to twenty, and it is not very long before their teeth begin to decay. Whether it is a climatic effect or change of habits, I am unable to say. We ought to take more pains in following out the history of such cases.

*Dr. Field.*—Another point we have to consider in this matter of immigration is the effect of change of climate. While practising in London I had an opportunity of observing the bad results that I believe to be due to change of climate and modes of life. A great many men are sent out from England to India, Africa, and Australia, to do government service for varying terms of years in those countries, and in most cases the effect of the climate seems to produce more or less retrogression in the condition of the teeth, and it was understood in the office that everything possible should be done for such patients to prevent the inevitable inroads of decay, for, even with the best care they could get, they would come back in a bad state. If I were to give an opinion, I would say that the retrogression in the quality of teeth was due to a change of climate or change in the habits of life, rather than to the intermingling of races.

*Dr. Reilly.*—If I were to express my opinion, it would be to endorse Dr. Blaisdell, and attribute it wholly to the food and change in the manner of living. I think if you take the same grade of civilization and occupation of any of the races, that you will find the same conditions of the teeth. The domestics that we get, a great many of them, come to this country from seventeen to twenty years of age, and enter domestic service. Previous to that many of them have done nothing, except possibly to assist about the house. As to the field life, I think that is a thing of the past, at least in Ireland. The women and girls do such work about the farm-house as you will find them doing in New England, but to go out into the field and follow the plough and harrow, etc., I think there is very little of that done in Ireland to-day. It may be done in Germany and Sweden, but in Germany you will find just as severe ravages among the teeth of the upper classes as you will find among the peasants.

At the time of the building of the subway, most of the laborers employed in the work were Italians. There were some Irishmen, who generally held some advanced position, such as the boss of a gang. Quite a section of it was built in front of my office, and I could look out of the window and see the workmen eating their dinners, and it was interesting to see the difference between the Italian's and the Irishman's dinner. Some of the Italians had dinner-pails, but in many cases, almost universally, the Italian at the noon hour would go to his coat on the bank and fish out a long roll of dry bread and break it into pieces and sit there munching it without drinking anything with it, at least while he was eating. With the Irishmen, in many cases the wife would come with a large pail, sometimes with one or two children accompanying her, and the foreman would go off under some tree, spread a napkin on the grass, open the pail, and go at the food with knife and fork; and oftentimes the children would squat down and partake of the dinner. Now, in my mind the teeth of the Italian would outlast those of his boss, because the Italian used his teeth more.

As to girls in domestic service, I have done work for a great many of them. I have questioned many of them, and they tell me that they are tasting and nibbling food all day long, especially kitchen girls, and of course you cannot expect them to have clean teeth under those circumstances.

As far as the climatic effect is concerned, I do not think there is anything in it. I judge it to be wholly a question of food and care. If the teeth are cared for and exercised, I think they would be better in quality.

For a long time I have had charge of the teeth of some cloistered ladies in a religious institution, where they eat no meat at all. I do not imagine that they take soups. Their diet is of the plainest kind,—the different kinds of bread, vegetables, fish, milk, and a very little pastry. Of course such food does not require a great amount of mastication, but those ladies have excellent teeth. They care for their teeth, they have been instructed to, and they are under dental care all the time; at least their teeth are examined twice a year, and they have as fine teeth, I think, as the same class of people outside.

That brings up a little matter in connection with my own family,—the contrast in the quality of the teeth of my boy, fourteen years of age, and a girl of fifteen. The girl has always been healthy from birth. A year ago I put in fifteen cement fillings for her. The boy, up to the age of eight or nine was very delicate. The first year of his life we never thought we could pull him through, as he suffered severely with indigestion or malnutrition, and was at death's door several times. Within the past few years he has had but two or three permanent teeth filled. I looked for the boy's teeth to be poor, but he has excellent ones. I cannot explain this difference. Perhaps a remote explanation might be found in heredity, as the teeth of all the members of my wife's family are very poor; on the other hand, the teeth in my family are very good, better than the average. It cannot be the care, for I try to see that they are cared for equally well. If anything, the girl takes the better care of her teeth. A peculiar thing about it is that if the girl should neglect to brush her teeth for a day or two, I can detect it across the table, but I cannot with the boy.

*Dr. Field.*—In my remarks with regard to climate I did not mean to convey the impression that I considered change of diet or change of habits had no effect upon the condition of the teeth, because I do believe that any change which affects the system, such as change of food, habits of life, occupation, climate, or any other change which will produce an altered condition of the system, will also have its effect upon the teeth.

*Dr. Holmes.*—I do not see how we can always attribute the condition of the teeth to the food eaten. The Chinaman has pretty good teeth, and his diet is principally rice; possibly a few times a week he will take meat. The South Sea Islander has excellent teeth. His diet is a substance called poi, of a very thin consistency. They designate it as one-finger, two-finger, or three-finger poi, as it can be taken up by one, two, or three fingers from the dish. Their teeth have no work to perform so far as masticating their principal diet is concerned. Then there is the Scotchman. Many of them live principally on a sort of porridge that requires very little masticating. It has been the custom to place a good deal of stress on the influence of the Scotchman's porridge, as well as the coarse breads, etc., in the nourishing of teeth and building them up, but I have seen it stated on good authority, and as the result of many years' experience, that more benefit was to be derived by the teeth from a general vegetable and meat diet than by eating too much coarse bread; in fact, that in a general diet, what we call white bread would serve its purpose better than the coarse bread, because, in trying to assimilate the coarse bread which was taken into the system with a general diet of vegetables and meat, the best results from the whole were lost. If a wholly bread diet is to be depended on, the coarse bread is better; but living as we do, on quite a varied menu, the bread as prepared to-day, from a finely pulverized flour, is more thoroughly assimilated.

*Dr. Dickerman.*—I think Dr. Holmes is mistaken when he states that the Chinese live almost entirely on rice. A resident of Shanghai told me that the Chinamen used his rice in the same way as we do bread; that is, that it appeared at every meal, and in many cases they may make a meal of it; but the Chinese are great pork-eaters, and where they can afford it, fowl, ducks, geese, chicken, and the various meats and vegetables. The rivers in China near the large cities are sometimes completely covered with duck-farms, which supply the wants of the people.

About the South Sea Islander, I think you will also find that considerable pork and fish are included in his diet, and that they cannot be said to be entirely dependent on poi.

*Dr. Holmes.*—I would say that what I intended, in referring to the rice as used by the Chinamen, and the poi as used by the South

Sea Islander, was to show not only that a diet might not include much animal matter, but that the teeth had but little work to do in the eating. There is a good deal of stress laid sometimes on the work that we give our teeth to do, as though that were a great factor in their preservation, so I just simply mentioned that to show that in some cases the food that is the principal article of diet of a people does not give their teeth much work to perform.

*Dr. Blaisdell.*—Speaking of the teeth of the Chinese and Japanese, I have never seen any of that class who act as laundrymen, but I have seen a number of those who serve on board our government ships in the capacity of stewards, etc., and for cases of pyorrhœa, I think the Americans cannot compare with them. I really dread to see one of them come into my office. I have never seen an old Chinaman or Japanese, and out of curiosity I would like to see what would be the condition of their teeth in old age. I remember seeing one or two in California that were old, but they were minus teeth.

*Dr. Dickerman.*—The class of foreigners that I have come mostly in contact with, and those of foreign-born parentage, are Portuguese and French Canadian. The Portuguese come here and perform manual labor either in the brickyards, or on sewer gangs, street gangs, and what not, or become farmers, and their children either help on the farms or go into the cotton-mills. Those people seldom marry outside of their race, and are particularly clannish. Of course, in the seaport towns some go to sea, but there is a good deal of difference in the teeth of those people who have the outdoor life and those in the cotton-mills. Whether the girls are in the habit of using snuff or not, I do not know, but the teeth look very much like it. Still, that is more a question of environment. But take, for instance, the teeth of the grandfather, father, and daughter, and you can see a distinct change from one generation to the other, not only in the number of cavities, but in the size and shape of the teeth. The molars seem to grow more rounded instead of the large, heavy, square teeth, and the third molars get smaller and more crowded and are of no use at all, and it seems to me that, taking those teeth where you can see the three generations, it forms quite an interesting study.

*Dr. Reilly.*—I would like to ask Dr. Dickerman regarding the condition of the teeth of snuff-users. Why I ask is because, in my old home, Lowell, which is a manufacturing town, the mill operatives there are almost universally addicted to that habit, and whenever a few of them get a spare moment, it is devoted to a "scour." They take a waste rag, wrap it around the finger, wet it and dip it into the snuff, and scour away at the teeth; and they may be seen any noon hour in little squads on the banks of the river, and summer evenings in the fields, doing that. Since then I have often wondered what effect that had upon the teeth. It is a habit that has been imported from England, from the manufacturing communities there.

*Dr. Dickerman.*—The cotton mills in Taunton, where I live, make the higher grades of dress cloths, and there is not so much of that done as in the mills that do their own spinning. In Fall River, I think, every corner store has a pyramid of snuff-cans in the window, and they sell thousands and thousands of ounces annually. I have noticed that the spinners who come to Taunton from Fall River usually have a broad black line right under the gum. Many of them have pyorrhœa, which is no doubt caused by the constant irritation. I do not think they use waste in Fall River. They usually take two or three wooden toothpicks and chew the ends, which makes a sort of brush, and dip that into the box and brush their teeth with it, and of course those wood fibres get down under the gums and make bad work. The gums are invariably soft and bleed easily, and it makes a filthy mouth.

*Dr. Giblin.*—I cannot add anything further than to corroborate what has been said. I believe that change of habit and food often produces considerable havoc in the condition of the teeth of immigrants. Certainly I have myself seen cases of both sexes who have come to this country with magnificent teeth, and that shortly after coming here rapid decay of the teeth has occurred which has been very discouraging to both patient and dentist in attempting to control. I am inclined to believe that this is altogether consequent upon their change of food and in their social environment.

HARRY WEST HALEY,
*Editor Harvard Odontological Society.*

